# The first outbreak of African swine fever in Sweden: a survey of pig farmers' perceptions of information received, risks, biosecurity measures and future prospects

**DOI:** 10.1186/s13028-023-00722-w

**Published:** 2023-12-18

**Authors:** Elisabeth Rajala, Hedvig Gröndal, Susanna Sternberg Lewerin

**Affiliations:** Division of Bacteriology and Food Safety, Department of Biomedical Science and Veterinary Public Health, Swedish University of Agricultural Sciences, P.O. Box 7054, 750 07 Uppsala, Sweden

**Keywords:** ASF, Control, Impact, Pig production, Wild boar

## Abstract

**Background:**

African swine fever (ASF), a viral hemorrhagic disease in domestic pigs and wild boar with up to 100% case fatality, was confirmed in Swedish wild boar in September 2023. The responsible authorities launched a control programme to eradicate the infection. The aim of the current study was to understand (i) how Swedish pig farmers have perceived the information issued by authorities and other stakeholders since the discovery of ASF in wild boar, (ii) which risks they see for introducing the infection to their farm, (iii) what biosecurity measures they have taken on their farms, and (iv) their outlook on the future. Such information is important for evaluating the effectiveness of the early stages of ASF control in Sweden. A questionnaire was designed and distributed to members of the Swedish pig producers’ organisation.

**Results:**

A total of 155 farmers responded to the survey (response rate 36%). Almost all respondents had received general information about ASF (91%, n = 138), and 72% (n = 109) had received information about how they can protect their farm from ASF introduction. A majority (87%, n = 118) thought the information was easy to understand, 90% (n = 137) that is was relevant, and 77% (n = 117) that they currently did not lack any information. If given the resources necessary, 58% (n = 84) of the farmers would like to take additional measures such as fencing, and heavily reduce or eradicate the wild boar population. Wild boars were considered the greatest risk for introduction of ASF into their herd (39%, n = 57), followed by people (30%, n = 44), and transports (16%, n = 23). Many farmers (66%, n = 88) had a positive outlook on the future, and 89% (n = 127) have not changed their plans for the future since the ASF outbreak.

**Conclusions:**

The responding farmers were in general satisfied with the information received in the beginning of the ASF outbreak. The majority have a positive outlook on the future and the outbreak has not caused them to change their plans. Actions that were highlighted as important to safeguard Sweden's pig production included measures to control the wild boar population.

**Supplementary Information:**

The online version contains supplementary material available at 10.1186/s13028-023-00722-w.

## Background

African swine fever (ASF) is a viral hemorrhagic disease in domestic pigs and Eurasian wild boar with up to 100% case fatality [1]. The disease was first described in East Africa, where the virus spread asymptomatically in the warthog population [2]. ASF is easily spread among pigs and has significant impact on the pig industry, leading to economic losses and disruptions in pig farming and the entire pig value chain in affected countries [3]. After years of persistence only in Africa and on the island of Sardinia, the disease is currently present in most regions of the world [4]. Since 2007, when the disease was first detected in Georgia [5], ASF has spread through Europe, and several European countries are currently affected [6]. In 2022, eight countries within the European Union (EU) (Bulgaria, Germany, Italy, Latvia, Lithuania, Poland, Romania and Slovakia), and four neighbouring countries outside of EU (Moldova, North Macedonia, Serbia and Ukraine) reported ASF outbreaks in domestic pigs [7]. The proportion of outbreaks was highest in Romania, which accounted for 87% of all outbreaks within the EU. A total of eleven EU countries reported outbreaks in wild boar, the same eight countries with outbreaks among domestic pigs, and three additional countries (Czechia, Estonia and Hungary). Outbreaks in wild boar were also reported in four neighboring countries outside of EU (Moldova, North Macedonia, Serbia and Ukraine) [7]. The transmission routes for the ASF outbreaks in Europe have mainly been direct or indirect contact with infected wild boar or domestic pigs, or by ingesting contaminated materials [8, 9].

**Fig. 1:**
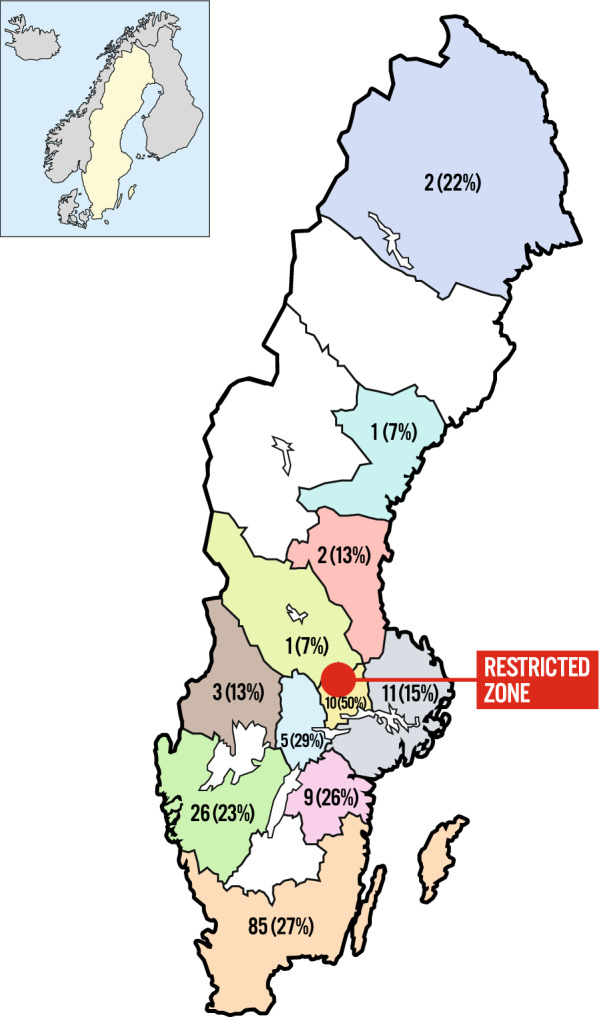
The geographical distribution of the respondents. The number represent the total number of respondents in each region, and the percentage indicates the number of farmers who responded in relation to the total number of pig farmers in the area

On the 6th of September 2023, the first case of ASF was confirmed in Swedish wild boar [10]. All cases were clustered in one defined area, suggesting a point-source introduction. A restricted zone of 1000 km^2^ was established, where all forms of activity, forestry, agriculture, hunting, and the right of public access outside main roads was banned (Fig. 1). In total, 22,000 people including 5000 landowners live within the restricted area. By October 2023 49 wild boars in total have been confirmed infected with ASF virus, all within the core area of the restricted zone, with no signs of spread of the virus outside the restricted zone [11]. The initial control strategy focused on searching for carcasses inside the core area, sampling and safe destruction of these. Subsequently, fences have been put up around the area where infected carcasses were found and possibly hunting will be implemented outside this area. However, there are also measures that are important outside the restricted zone, to prevent the spread and to detect any introduction of the virus in other parts of the country. All pig keepers have been advised to review their biosecurity measures and reminded to contact a veterinarian if there are signs of disease or increased mortality among the pigs [10]. Farmers play a key role in the control of contagious animal diseases, and their behaviour in the context of disease risk management in pigs [12, 13], poultry [14], and cattle [15, 16] has been studied. A recently published study from Germany [16], investigated pig farmers’ decision-making concerning biosecurity measures against ASF. The study found that most pig farmers did not perceive an increased threat to their farms, but were unsure how to properly implement biosecurity measures in accordance with the law.

The aim of the current study was to gain a better understanding of (i) how Swedish pig farmers have perceived the information issued by authorities and other stakeholders since the discovery of ASF in wild boar in Sweden, (ii) which risks they see for introducing the infection to their own farm, (iii) what biosecurity measures they have taken on their farms, and (iv) their outlook on the future. Such information has not been published previously but is important for evaluating the effectiveness of the early stages of ASF control in Sweden.

## Methods

### Study area and study design

The target population was Swedish pig farmers who are members of the Swedish pig producers’ organisation “Sveriges Grisföretagare” [18]. Out of Sweden’s 1100 pig farms [19], approximately 600 are members of the pig producers’ organisation [18]. The aim was to enroll as many farmers as possible in the survey, within a short period of time, but with no specific numerical target.

### Study procedure

A questionnaire was designed in Netigate with 17 questions on farm characteristics, information related to the ASF outbreak, biosecurity measures implemented, and future prospects for pig production. The questionnaire was pre-tested on three pig farmers to allow for improvements. The questions were either open-ended, multiple-choice or single-choice. A translated version of the questionnaire is provided in Additional file 1: S1. Responses were completely anonymous and participation was voluntary. No data regarding the identity of the farmers were collected in order to make sure that information obtained from the survey could not be traced back to the individual respondents. The questionnaire was distributed by the pig producers’ organisation to all their members via email on the 18th of September 2023, a reminder was sent out on the 2nd of October, and the survey was closed after 3 weeks on the 9th of October 2023. Descriptive statistics were used to summarise the results of multiple-choice questions. The responses to open-ended (free text) questions were categorized based on thematic analysis.

## Results

### Farm characteristics

A total of 157 farmers responded to the survey which corresponds to approximately 14% of all pig farmers in Sweden, and the response rate was 36%. Answers from two of the respondents were removed from the analysis due to the fact they were not pig producers, resulting in 155 participants. The geographical distribution of the respondents is illustrated in Fig. 1. The majority (88%) of the respondents kept their pigs strictly indoors, while 6% kept their pigs both indoors and outdoors. Three percent kept the pigs indoors with some outdoor access, and 3% kept the pigs only outdoors. In total, 105 respondents kept sows, the median number of sows was 300 (range 12–3150). Of the 122 respondents keeping slaughter pigs the median herd size was 1600 (range 20–14,500).

### Information related to the ASF outbreak

In total, 136 to 151 farmers responded to the question about ASF-related information. Almost all respondents had received general information about ASF (91%), and 72% had received information about how they can protect their farm from ASF introduction. One-third (31%) of the respondents had received more specific requirements or recommendations on biosecurity measures that they need to implement on the farm. The follow-up question, if the information was easy to understand, had 136 respondents of which 87% answered “yes”, 16% answered “partly” and only 2 respondents (< 1%) answered “no”. Another follow-up question was if they thought the information received was relevant. A majority (90%) ticked “yes”, 13% answered “partly” and none of the respondents answered “no”. Comments from the two respondents that answered “partly” was that “they already knew everything/had received information” and “it is difficult to implement some recommendations”.

The farmers were also asked “Is there any type of information on ASF that you currently lack”? The majority (77%) of the farmers answered “no”. Eleven per cent answered “partly”, and 11% answered “yes”. Twenty one of the farmers that answered “yes” or “partly” stated that they would have liked more information on (1) how to put up fences, (2) how the municipalities will prevent wild boar from entering city dumps, (3) how to handle animal- and feed transports close to the restriction area, (4) wild boar movements within the restricted area, (5) how to use straw as bedding material in a safe way, (6) how to handle food waste in the nature (targeting the public), and (7) daily updates to the farmers on the Swedish ASF situation.

The last question in this section was “What has been your main source of information since the outbreak started”? The majority (32%) answered “my veterinarian”, 18% answered “the Swedish board of Agriculture”, 16% answered The Federation of Swedish Farmers (LRF), 16% answered “my animal health organisation”, and 14% answered National Veterinary Institute (SVA).

### Biosecurity measures implemented

In total, 145 farmers responded to the questions on biosecurity measures (Table 1).

**Table 1:** Questions linked to biosecurity answered by Swedish pig farmers (n = 145)

Question	Category	n	%
Have you changed any biosecurity measures in your herd since the ASF outbreak?	Yes	38	26
	Partly	37	26
	No	70	48
Have you been able to implement the recommended measures to prevent infection?	Yes	83	57
	Partly	34	23
	No	8	6
	I have not received information about biosecurity measures	20	14
In addition to the measures you have already taken, are there other measures you would like to take to reduce the risk of infection (if you were given all the resources required)?	Yes	84	58
	No	61	42
What do you see as the greatest risk of introducing infection into your herd (you may choose more than one)? (open-ended)	Wild boars	57	39
	Employees/visitors /veterinarians	44	30
	Transports	23	16
	Straw	16	11
	Recruitment of new pigs	5	3
	Other or don’t know	19	13

A majority (52%) of the farmers ticked “yes” or “partly” on the question if they had changed any biosecurity measures since the outbreak of ASF, whereas 48% had not changed their biosecurity measures. Among the farmers that had changed biosecurity measures since the ASF outbreak, some measures implemented were (1) washing clothes in warmer temperatures (60 °C), (2) improved procedures for change of footwear, (3) no visitors in the stables, (4) built temporary hygiene locks, (5) disinfection of tyres on farm vehicles, (6) closed some entrances to the farm and put up surveillance cameras, and (7) put up or improved electric fencing. Another comment from a farmer was that “we are discussing how to increase biosecurity around the loose housing and introduction of bedding materials but have not found a solution yet”.

A follow-up question was if they had been able to implement the recommended measures to prevent infection where the majority (57%) of the respondents ticked “yes”. Of the respondents that answered “partly” or “no” some of the reasons were (1) not had time to put up fences yet, (2) public road runs right through the farm, (3) the machine used for emptying bedding is still a risk factor, and (4) difficult to safely remove litter from deep straw beds. Another comment was that the current outbreak is quite far away and there are so many farm duties during this particular time period so there is not time nor energy to implement all recommendations that you would have wished.

Another question in this section was “In addition to the measures you have already taken, are there other measures you would like to take to reduce the risk of infection (if you were given all the resources required)?” The majority of the respondents (58%) answered “yes”, and of those there were two measures dominating the answers which were “fencing” (n = 50, 60%), and “heavily reduce or eradicate the wild boar population” (n = 9, 11%).

The final (open-ended) question in this section was “What do you see as the greatest risk of introducing infection into your herd?” The presence of wild boars was considered the greatest risk (39%), followed by people (30%), and transports (16%).

### Future prospects for pig production

The last section of the survey focused on future prospects for the pig production, and 133 respondents answered the open-ended question “How do you see the future for the pig production”. The majority (66%) were positive and thought the future looked bright. Sixteen percent expressed concerns about the production, and 13% expressed neither optimism nor pessimism. Eight farmers (6%) answered that they plan to discontinue production in the next few years. A follow-up question was “Have your plans for the future changed since the ASF outbreak”. The question was answered by 142 farmers of which 11% (n = 16) answered “yes”, and 89% (n = 127) of the respondents answered “no”. Of the farmers that answered “yes” the most common reason mentioned was that they will put a hold on new investments. The final question in this section addressed if the farmers* “*Would be able to change or diversify the production if necessary, and in what way”? The majority (71%) of the 82 respondents answered “no”, 11% answered “don´t know”, and 17% had some idea about how to change the production. Examples of changed or diversified production included (i) switch to crop production, (ii) switch to poultry or egg production, (iii) change to indoor pig production, and (iv) reduce the current pig production.

### Other comments

At the end of the survey, the respondents were given the opportunity to give free comments. Seven farmers expressed that they appreciate the information given from the authorities and some of them were also impressed with how the authorities have handled the ASF outbreak so far. Seven farmers highlighted that the wild boar population in Sweden needs to be better controlled, and three farmers expressed concern that wild boars have access to city dumps, or food waste, in some municipalities. Two farmers wished for financial support to implement specific biosecurity measures on their farms.

## Discussion

This survey shows that the respondents in general are satisfied with the information they received in the beginning of the ASF outbreak, that they share common views and concerns on potential risk factors, with wild boars considered to be the major threat, and that the majority still have a positive outlook on the future.

The fact that farmers responded to the survey within a short time frame suggests that they found the survey relevant and that they appreciate being able to make their voice heard. Although the median herd sizes were slightly larger than the average pig herd size in Sweden, herd demographics of the respondents reflected the Swedish pig population. The majority of the farms kept their pigs only indoors which is in accordance with the structure of Swedish pig production with few organic farms (less than 3% of the total pig population in 2021) where outdoor production is mandatory [20].

Almost all respondents had received general information about ASF, and the majority (72%) had also received information about how they can protect their farm from ASF infection, which is encouraging as knowledge is a key factor in animal disease management on farms [17, 21, 22]. Also, almost all respondents thought the information was relevant and easy to understand. The level of awareness seems to be higher among the Swedish pig farmers compared to a German study where around a third of the farmers thought they knew enough about ASF [17].

However, only one third of the respondents in the current study had received more specific requirements or recommendations on which biosecurity measures they should implement on the farm. One reason could be that authorities and veterinarians think that the general recommendations communicated are sufficient and that specific ones are not needed. Another possible explanation is that the authorities, and farm veterinarians consider that the level of biosecurity in Swedish pig production in general is sufficiently high and that farmers who follow the standard recommendations and regulations are well protected against ASF infection. The follow-up question if the farmers currently lack any information, showed that only one quarter of the farmers would like more specific information such as how to put up fences, how to handle transports close to the restriction area, and also more general information on the control efforts, e.g. how the municipalities will address the issue of the dense wild boar population, or how to avoid wild boar accessing food waste or city dumps. This indicates that the respondents felt they had received sufficient information about ASF and, as previously found, are well aware of the fact that the dense wild boar population in many counties is considered to be an important risk factor for ASF [23].

The farmers reported that they used various information sources such as their farm veterinarian, their industry organisation (LRF), and the veterinary authorities. The importance of veterinary officials and farm veterinarians has also been acknowledged in a study targeting German pig producers, concluding that it is important to focus on joint decision-making and take farm-specific circumstances into account when discussing biosecurity measures [17]. Also, a study from England showed that sheep and pig farmers considered vets as the prime source to interpret generic advice from national bodies, because veterinarians have a good knowledge of the local context [24].

The farmers were also asked about the greatest risk factor for transmitting ASF virus to the individual farm. There were three factors dominating the answers; wild boars were considered to be the greatest threat, followed by human visitors, and transports. A recently published study showed that 80% of the Swedish pig farmers saw wild boar activity in the vicinity of their farm at least once during the year [23]. This could explain why the respondents in the current study mentioned wild boars as an important risk factor, in addition to the fact that the disease had recently been detected in wild boar.

More than half of the respondents had improved the biosecurity on their farm since the ASF outbreak, such as prohibited visitors from entering the stables, improved procedures for changing footwear, and put up or improved electric fencing on the farm. A majority of the respondents had, at least partly, been able to follow recommendations on measures to prevent infection. This is in contrast to the German study where many pig farmers communicated their uncertainty on how to correctly implement biosecurity measures according to the law, and where less than a third of the pig farmers stated that a higher perceived risk made them improve their biosecurity measures [17]. Furthermore, an American study showed that one important factor influencing if the pig producers adopted biosecurity measures was the perceived feasibility in their operation [13]. Hence, the findings in the current survey suggest that the Swedish pig producers have both sufficient understanding and the means to implement the recommended measures. Other studies have also shown that farmers who implement biosecurity measures can serve as an incentive for other farmers to do the same [25]. However, whether information sharing was a factor influencing the willingness to strengthen biosecurity measures also among Swedish pig farmers was not investigated in the current survey. Reasons for not implementing the recommendations given by the farmers in the current study included time constraints, heavy workload, geographical distance from the outbreak, and public roads preventing fencing around the farm. That farmers perceive time constraint as a barrier to implementation of biosecurity measures has been shown in an interview study with English pig and sheep farmers. The English study also found that perceived geographical isolation might lead to a perception that biosecurity measures were unnecessary [24].

Many farmers indicated a wish to implement further measures if they had the means to do so. The two most commonly described measures were “fencing”, and “heavily reduce or eradicate the wild boar population”. This reflects experiences from the ASF outbreaks in Europe where infected wild boars were the dominating risk factor for spread and persistence of the disease [26], and fencing has been used as an important part of successful eradication [27].

The majority of the farmers had a positive outlook for the future and their pig production. In contrast to the Swedish pig producers in the present study, Alcaron et al. [22] showed a negative and pessimistic perception among English pig producers about the current economic situation of the industry and of their farms. Almost 90% of the farmers in the present survey expressed that the ASF outbreak had not influenced their production plans, and only a few farmers expressed concerns for their production. Similar findings were reported in a study from Germany [17]. This may indicate that Swedish pig farmers have confidence in the authorities, and that they believe that the ASF eradication will succeed.

The majority of the farmers stated that they would not be able to change their production system. This question aimed to assess the possibility for adapting the pig production if ASF would become established in the wild boar population in an area. The result is consistent with the current farming systems in the industrialised world being highly specialised with few opportunities to diversify the production if necessary [28].

In the current study potential biases could have arisen if the questions were interpreted incorrectly by the farmers, or due to self-selection bias. However, the questionnaire was pre-tested to allow for improvements, and 36% of the invited farmers choose to participate within a short timeframe. It might be expected that the respondents were farmers who felt more strongly about the issue, in a positive or negative way. The overall positive attitude of the respondents was therefore somewhat surprising. The collaboration with the farmers’ own organisation was intended to achieve a high response rate but may also have skewed the response towards farmers with a stronger interest in the future of the pig industry. For the purpose of this study, such a skewness would not necessarily be a problem. We therefore consider the results to give a reasonably representative picture of pig farmers' perceptions of information received, risks, biosecurity measures and future prospects.

## Conclusions

The responding Swedish pig farmers were generally satisfied with the information they received in the beginning of the ASF outbreak. A majority of the respondents had, at least partly, been able to follow recommendations on measures to prevent infection. Most of the responding farmers have a positive outlook on their future pig production and the outbreak has not yet caused them to change their plans. Actions that were highlighted as important to safeguard Sweden's pig production in the future included a drastic reduction of the wild boar population, and ensuring that wild boar cannot come into contact with food waste.

### Supplementary Information

**Additional file 1.** Questionnaire.

## Data Availability

All data are available from the corresponding author upon reasonable request.
