# Supplementary material for: The first outbreak of African swine fever in Sweden: a survey of pig farmers' perceptions of information received, risks, biosecurity measures and future prospects
Source: Acta Vet Scand. 2023 Dec 18;65:58. doi: 10.1186/s13028-023-00722-w (PMC10729408; doi:10.1186/s13028-023-00722-w)
Supplement: Supplementary file 1 — Additional file 1. Questionnaire. [file 13028_2023_722_MOESM1_ESM.pdf]

Questionnaire for Swedish farmers about the swine fever outbreak (translated from Swedish)

This questionnaire is sent to members of Sveriges Grisföretagare (the Swedish pig farmers' organisation). The objective is to gain a better understanding of how you have perceived the information provided by different governmental and non-governmental actors since the detection of African swine fever in wild boar in Sweden, what actions you have taken on your farm and how you see the future. The responses are totally anonymous and responding will take a maximum of 10 minutes. Your views and experiences are valuable to obtain a current picture of the situation of Swedish pig farmers. The results will be used in research and communicated by Sveriges grisföretagare.
We would be grateful for your responses before 30th September. Thank you very much for your participation!

Farm information

1. Production type (multiple-choice, one option possible)

- The pigs are kept strictly indoors
- The pigs are kept indoors with possibility for outdoor contacts (e.g. via loose panelling)
- The pigs are kept strictly outdoors
- If nothing of the above alternative apply, please specify ___________________

2. What county is your farm situated in? (multiple-choice, one option possible)

- Blekinge län
- Dalarnas län
- Gotlands län
- Gävleborgs län
- Hallands län
- Jämtlands län
- Jönköpings län
- Kalmar län
- Kronobergs län
- Norrbottens län
- Skåne län
- Stockholms län
- Södermanlands län
- Uppsala län
- Värmlands län
- Västerbottens län
- Västernorrlands län
- Västmanlands län
- Västra Götalands län
- Örebro län
- Östergötlands län

3. Please provide the number of sows that can be kept in your herd (in figures). Write 0 if you don't have any sows. _______________

4. Please provide the number of fattening pigs that can be kept in your herd (in figures). Write 0 if you don't have any fattening pigs. ____________

Information prompted by the outbreak

1. What type of information have you received from the authorities and others (e.g. animal health organisation, Farmers' Association, herd veterinarian etc) since the outbreak of African swine fever in Fagersta? (multiple-choice, multiple options possible)

- General information about African swine fever
- Information about how to protect your herd from the infection
- Requirements or recommendations about what actions you need to implement on your farm
- Other information, please specify:_________________

2. Was the information easy to understand? (multiple-choice, one option possible)

- Yes
- No
- Partly

If you answered No or Partly, please explain why ______________

3. Was the information relevant? (multiple-choice, one option possible)

- Yes
- No
- Partly

If you answered No or Partly, please explain why _______________

Information about the outbreak

1. Is there any information about swine fever that you currently lack? (multiple-choice, one option possible)

- Yes
- No
- Partly

If you answered No or Partly, please explain why _______________

2. What has been your most important source of information since the beginning of the outbreak? (multiple-choice, one option possible)

- My animal health organisation
- The Swedish Farmers' Federation
- The Swedish Board of Agriculture
- The National Veterinary Institute
- My herd veterinarian in
- Other, please specify _________________

Actions taken since the outbreak of African swine fever

1. Have you changed any biosecurity routines on your farm since the outbreak? (multiple-choice, one option possible)

- Yes

- Partly
- No

If you answered Yes or Partly, please describe the actions ________________

2. Have you been able to implement the actions that have been recommended to prevent introduction of the infection to your farm? (multiple-choice, one option possible)

- Yes
- Partly
- No
- I haven't received any information about actions

If you answered Partly or No, please specify why ____________________

3. In addition to the actions you have taken, are there other measures that you would like to implement to reduce the risk of introducing the infection (if you were given unlimited resources) (multiple-choice, one option possible)

- Yes
- No

If you answered Yes, please describe briefly ________________

4. What do you see as the greatest risk of introducing infection into your herd?

____
____
____

Future prospects for pig production

1. How do you see the future of your own pig production?

____
____
____

2. Have your plans changed since the outbreak?

- Yes
- No

If you answered Yes, please describe briefly _____________________________

3. Would it be possible to change your production if needed and, if so, how?

____
____
____

Other comments

If you have any other comments, they are most welcome

____
____
____

Thank you very much for your participation!
